# 

**Published:** 1867-11

**Authors:** 


### CONTENTS FOR NOVEMBER.

ORIGINAL CONTRIBUTIONS.

Fibrous Polypus of Uterus—Removal by Ligature. By G. W. Phillips, M.D., Dixon, Ill., ---- 513

Spinal Irritation. By C. F. Hart, M.D., ---- 526

Opium and Belladonna. By James T. Neuman, M.D., ---- 529

A Paper Read before the Chicago Medical Society. By Hiram Wanzer, M.D., Chicago, Ill., ---- 532

The Endoscope, and its Application to the Diagnosis and Treatment of Urinary Affections. By A. J. Desormeaux, M.D., Translated by R. P. Hunt, M.D. Continued from Page 464 ---- 545

FOREIGN CORRESPONDENCE.

Extracts from a Private Letter from Prof. Freer, dated Stuttgart, Sept. 13, 1867, ---- 538

EDITORIAL.

The next Volume, ---- 542

Cliniques at Rush College, ---- 543

BOOKS RECEIVED.

The Practice of Medicine and Surgery, Applied to the Diseases and Accidents incident to Women. By Wm. H. Byford, A.M., M.D., ---- 543

A Biennial Retrospect of Medicine, Surgery, and their Allied Science. By Mr. H. Power, Dr. Anstie, Mr. Holmes, Mr. Thomas Windsor, Dr. Barnes, and Dr. C. Hilton Fagge, ---- 544

Inhalation, its Therapeutics and Practice. By J. Solis Cohen, M.D., ---- 544

Studies in Pathology and Therapeutics. By Samuel Henry Dickson, M.D. 544

Headaches: Their Causes and their Cure. By Henry G. Wright, M.D., ---- 544

Epidemic Meningitis, or Cerebro-Spinal Meningitis. By Alf. Stille, M.D., 544

Hufeland's Art of Prolonging Life. By Erasmus Wilson, F.R.S., ---- 544

Circular No. 7, ---- 544

SELECTIONS.

Physiological and Therapeutical Action of Cod Liver Oil, ---- 556

